# Preventive effect of *Lacticaseibacillus rhamnosus* 2016SWU.05.0601 and its postbiotic elements on dextran sodium sulfate-induced colitis in mice

**DOI:** 10.3389/fmicb.2024.1342705

**Published:** 2024-02-05

**Authors:** Linli Bu, Yang Li, Chen Wang, Yuhang Jiang, Huayi Suo

**Affiliations:** ^1^College of Food Science, Southwest University, Chongqing, China; ^2^Modern “Chuan Cai Yu Wei” Food Industry Innovation Research Institute, Chongqing, China

**Keywords:** DSS, NF- kappa B, postbiotic, probiotic, ulcerative colitis

## Abstract

Microbial-based therapies are one of the hotspots in the field of ulcerative colitis research. The lactic acid bacteria and their postbiotics occupy a key position in microbial therapies, however, the mechanism by which they alleviate ulcerative colitis in mice is unknown. We investigated the effects of *Lacticaseibacillus rhamnosus* 2016SWU.05.0601 (Lr-0601) and its postbiotics on male Kunming mice with dextran sulfate sodium salt (DSS)-induced ulcerative colitis (UC). The results showed that Lr-0601 significantly alleviated the deterioration of UC and restored the expression of intestinal mechanical barrier proteins. In addition, Lr-0601 significantly reduced the expression of inflammatory cytokines in the body and regulated the expression of key regulatory genes of the NF-κB-iNOS/COX-2 signaling pathway in colon tissues to a large extent. Our results suggest that supplementation with Lr-0601 and its postbiotics can effectively prevent DSS-induced UC and have a beneficial effect on intestinal health, which also provides new insights and research bases for the prevention as well as the treatment of ulcerative colitis and other diseases related to intestinal barrier dysfunction and other diseases.

## Introduction

Ulcerative colitis (UC), a non-specific chronic intestinal disease, mainly occurs in the sigmoid colon and rectum and is typically accompanied by abdominal pain and bloody diarrhea (Eisenstein, 2016; Ungaro et al., 2017). UC is characterized by a long course of disease, repeated attacks, difficulty healing, and has a strong tendency to become cancerous. The etiology and pathogenesis of UC have not been fully elucidated, but existing studies have concluded that UC results from a combination of genetic susceptibility, environment, infection, and abnormal immune response (Iyer and Corr, 2021). Currently, the treatment of UC mainly relies on traditional drugs such as amino salicylic acid, corticosteroids, biologics and immunosuppressants. Although they have clinical efficacy, they also have the disadvantages of high side effects and easy to form drug dependence (Yang et al., 2022). Therefore, it is of great practical significance to find new strategies for the prevention and treatment of UC that are safe and effective with few side effects.

Clinical studies and animal experiments have indicated that the occurrence of UC is closely related to intestinal flora imbalance (Yu, 2023). Research has also suggested that the core pathological feature of UC is loss of integrity of the intestinal barrier, resulting in increased bacterial antigen translocation and dysregulation of the intestinal flora. This increase promotes pro-inflammatory and inflammatory substances into the lamina propria of the intestinal mucosa, triggering an uncontrollable vicious cycle of immune response and leading to colonic damage (Capaldo et al., 2017). Intake of probiotics exerts positive effects on human intestinal health. The activities and metabolites of probiotics can improve intestinal health by inhibiting the growth of pathogenic bacteria, maintaining intestinal flora balance, and regulating intestinal barrier function and immune function. Thus, probiotics exhibit potential for the prevention and treatment of UC (Maciel-Fiuza et al., 2023). Whereas, when it comes to intake of probiotics, choosing natural food sources is more beneficial for body absorption and utilization and has a higher safety profile, therefore, *lactobacilli* strains isolated from yak yogurt were chosen for this study. It has been reported that various probiotics such as *Lactobacillus plantarum* HY01 isolated from yak yoghurt and *L. plantarum* CQPC06 isolated from kimchi have exhibited to prevent UC (Chen et al., 2017; Zhang et al., 2018).

Probiotic organisms and metabolites have been defined by the International Society for Probiotics and Prebiotics (ISAPP) in May 2021 as the term “postbiotic,” which refers to a substance that is beneficial to the health of the host. It refers to preparations of inanimate microorganisms and/or their components that are beneficial to the health of the host (Salminen et al., 2021). Recent studies have shown that postbiotics show potential in the treatment of UC, with some studies claiming that probiotics and postbiotics have similar abilities to improve disease phenotypes (Liu et al., 2023). For example, a preclinical study found that *Lactobacillus plantarum*-derived postbiotics were able to optimize the composition of the intestinal flora and significantly modulate the levels of short-chain fatty acids and neuroactive molecules (5-hydroxytryptamine, γ-aminobutyric acid) *in vivo*, which in turn ameliorated *Salmonella*-induced neurological dysfunction (Wu et al., 2022). In addition, researchers found that in a mouse model of colitis, postbiotics from *Bifidobacterium adolescentum* B8589 had a stronger gut microbiome-modulating effect than probiotics and were able to effectively attenuate colonic inflammatory cell infiltration, mucosal damage, and crypt loss and alleviate colitis symptoms in mice (Zhang T. et al., 2022). These studies suggest to us that postbiotics have antimicrobial, antioxidant, immunomodulatory and intestinal barrier capabilities, and have a greater potential to improve the health of the organism. However, current research has focused on the use of probiotics to alleviate intestinal diseases, while the *in vivo* application of postbiotics is still in its infancy, and their mechanism of action is still unclear. Therefore, the present study focused on the effects of probiotics and postbiotics on dextran sulfate sodium salt (DSS)-induced colitis. It also preliminarily investigates the mechanism of action of probiotics and postbiotics in improving colitis.

The strain Lr-0601 used in this study is a strain of lactic acid bacteria isolated from yak yogurt collected in Qinghai. Yak yoghurt, a fermented dairy product distributed in the Qinghai-Tibet Plateau, is prepared by local herders using traditional methods (Guo et al., 2013). Due to the unique geographical and climatic characteristics of the Qinghai-Tibet Plateau, the microorganisms in yak yogurt have formed a complex microbiota through natural selection and long-term domestication (Ding et al., 2022; Franceschi et al., 2023). Yak yogurt is richer in nutrients than regular yogurt and is considered a good reservoir of lactic acid bacteria (Wei et al., 2022), of which *Lactobacillus plantarum*, *Lactobacillus fermentum*, and *Lactobacillus swissii* are the dominant strains, and have a wide range of beneficial properties such as antioxidant and lipid-lowering (Zhang X. et al., 2022). Therefore, yak yogurt is an ideal source for the isolation and screening of excellent lactic acid bacteria. Lr-0601 is a strain of lactic acid bacteria isolated from yak yoghurt collected in Qinghai.

In the experiments presented in this study, we established a model of colitis by administering DSS and examined the effects of supplementation with Lr-0601 and its postbiotics in alleviating DSS-induced colitis. The findings confirmed that both probiotic and postbiotics alleviated symptoms associated with colitis, which was reflected in improved colon length weight, colon tissue status, *in vivo* inflammation levels, and intestinal barrier integrity in colitis mice. Our findings may help to capitalize on the potential benefits of Lr.0601 and its postbiotics in alleviating colitis, mitigating intestinal disease, and protecting intestinal health, and support that Lr-0601 and its postbiotics are promising and safe adjunctive therapies for colitis.

## Materials and methods

### Experimental strain

*Lacticaseibacillus rhamnosus* 2016SWU.05.0601 (Lr-0601) was isolated from traditional fermented yak yoghurt (Hexi Town, Guide County, Tibetan Autonomous Prefecture of Hainan, Qinghai, China), stained with Gram stain, subjected to 16s rDNA sequencing, and biochemical identification, and preserved in the China Center for Type Culture Collection (Wuhan, Hubei, China), with preservation number M2018592.

### *In vitro* tolerance assay of the strain

In simulated artificial gastric juice assay, 10 mL of Lr-0601 culture solution was centrifuged at 3,000 r/min for 15 min at 4°C. The collected pellets were washed with 10 mL of sterile physiological saline and resuspended in the same buffer. The resuspended cells and artificial gastric juice (0.2% NaCl, 0.35% pepsin, pH 3.00) were mixed at a ratio of 1:9 and incubated at 37°C for 3 h. The cultures were coated on MRS broth (BD, Franklin Lakes, NJ, USA) agar plates and incubated for 48 h at 37°C. The colony-forming units (CFU) on each plate were counted. Three parallels were made for each sample.

In the bile salt tolerance assay, Lr-0601 was inoculated in MRS broth plus 0.2% of sodium thioglycolate containing 0.00, 0.10, 0.20, and 0.30% (w/v) of porcine bile salt. The amount of inoculum was 2% (v/v). The mixture was then incubated at 37°C for 4 h. Bile salt tolerance was then conducted by reading the optical density of the bacterial culture solution at 600 nm. Three parallels were made for each sample.

### Animal grouping and administrations

All animal procedures described in this study were reviewed and approved by the Ethics Committee of Chongqing Medical University (Animal Experimentation Ethics: 201804021B). Fifty male mice, aged 6 weeks, from Kunming were purchased from Experimental Animal Center of Chongqing Medical University (Chongqing, China). The mice were exposed to the following conditions: temperature, 25°C ± 2°C; relative humidity, 50% ± 5%; light/dark cycle, 12 h/12 h. In addition, the mice were given free access to a standard chow diet and water. After a week of adaptive feeding, all mice were randomized into five groups (*N* = 10/group): normal, DSS, high-dose treatment with 1.0 × 10^10^ CFU/kg body weight (Lr-0601-H), low-dose treatment with 1.0 × 10^9^ CFU/kg body weight (Lr-0601-L) and the concentration of 1 × 10^10^ CFU/mL Lr-1601 bacterial suspension was heat-killed at 100°C for 30 min, centrifuged, and finally resuspended in physiological saline (Lr-0601-I). The experimental period was set to 5 weeks. Mice in the Lr-0601-H and Lr-0601-L groups were treated daily with Lr-0601 via gavage, mice in the Lr-0601-I group were treated daily with inactivated Lr-0601 at 1.0 × 10^10^ CFU/kg body weight via gavage, mice in the normal group and the DSS group were not administrated with lactic acid bacteria. The normal group were given free access to a standard chow diet and water from the beginning to the end of the experiment. The other mice were fed a normal diet but were given water with 2% (w/v) dextran sulfate sodium (DSS: 40 kDa; MP Biomedicals, Santa Ana, CA, USA) in Week 3 and 4% DSS in Week 5. The body weight of the mice was recorded daily during administration and the gavage volume was adjusted according to the body weight.

### Blood sampling collection and colon collection

Five weeks after administration, mice were fasted without water for 12 h. Blood samples were collected, transferred to 1.5 mL EP tubes, and then centrifuged at 3,000 rpm for 10 min at 4°C before serum was collected and stored in a −80°C refrigerator for further study. After obtaining blood samples, all mice were euthanised and the samples disposed of. Colon tissue was collected and the length and weight of the colon were quickly measured. About 1 cm of colon was taken, fixed in 10% formalin solution for 24 h, washed with water, dehydrated by alcohol gradient and embedded in paraffin, the tissue was cut into standard sections and stained with hematoxylin-eosin (H&E) for histological examination. The remaining colon tissues were stored in −80°C refrigerator for further study.

### Histological observations

After mice were executed, colon tissue samples were rapidly dissected and immersed in 10% neutral formalin solution, dehydrated with a gradient of 75, 85, 95, and 100% ethanol and embedded in paraffin wax for embedding. Then the wax blocks were cut into thin slices of about 5 μm and dried, then dewaxed by xylene, rinsed with ethanol and distilled water and stained with hematoxylin and eosin, and the stained sections were dehydrated by ethanol and transparent by xylene, then sealed with gum, and then observed and photographed under a microscope (BX43, Olympus Corp., Tokyo, Japan).

### Determination of cytokine levels in serum

Serum cytokine levels (IL-1β, IL-6, IL-8, and TNF-α) were determined using the enzyme-linked immunosorbent kit (Cloud-Clone Corp, Wuhan, Hubei Province, China).

### Quantitative real-time polymerase chain reaction

Total RNA in the colon tissue was extracted using Trizol Reagent (Invitrogen, Carlsbad, CA, USA) and then transcribed into cDNA by using RevertAid First Strand cDNA Synthesis Kit (Invitrogen, Carlsbad, CA, USA). The mixed PCR system was placed in a gene amplification instrument (A200, Langji Scientific Instrument Co., Hangzhou, Zhejiang Province, China) and run at 65°C for 5 min, 42°C for 60 min, and 70°C for 5 min for 40 cycles. Moreover, qRT-PCRs combining 1 μL cDNA, 1 μL Master Mix (Invitrogen, Carlsbad, CA, USA), 1 μL of forward primer and reverse primer (10 mmol/mL), and 7 μL ddH_2_O were conducted in an automatic thermocycler (StepOnePlus Real-Time PCR System; Thermo Fisher Scientific, Waltham, MA, USA), run for 40 cycles of 94°C for 30 s, 58°C for 30 s, and 72°C for 50 s and then at 75°C for 10 min. Relative mRNA transcription levels were calculated using the 2^–ΔΔ*Ct*^ method. The sequences of the primers used in this study are provided in Table 1.

**TABLE 1:** Sequences of primers used for qRT-PCR.

Primer name	Forward primer	Reverse primer
GAPDH	AGGTCGGTGTGAACGGATTTG	GGGGTCGTTGATGGCAACA
IL-1β	GAAATGCCACCTTTTGACAGTG	TGGATGCTCTCATCAGGACAG
TNF-α	CAGGCGGTGCCTATGTCTC	CGATCACCCCGAAGTTCAGTAG
ZO-1	GCCGCTAAGAGCACAGCAA	GCCCTCCTTTTAACACATCAGA
Occludin	TGAAAGTCCACCTCCTTACAGA	CCGGATAAAAAGAGTACGCTGG
IκB-α	TGAAGGACGAGGAGTACGAGC	TGCAGGAACGAGTCTCCGT
NF-κB	ATGGCAGACGATGATCCCTAC	CGGAATCGAAATCCCCTCTGTT
iNOS	GTTCTCAGCCCAACAATACAAGA	GTGGACGGGTCGATGTCAC
eNOS	TCAGCCATCACAGTGTTCCC	ATAGCCCGCATAGCGTATCAG
nNOS	CCCAACGTCATTTCTGTCCGT	TCTACCAGGGGCCGATCATT
COX-2	GGTGCCTGGTCTGATGATG	TGCTGGTTTGGAATAGTTGCT

## Statistical analysis

Data are presented in the form of means ± standard deviations. The statistical significance of differences among means was evaluated using ANOVA and Duncan’s multiple range tests; *p* < 0.05 was considered statistically significant. The analysis was performed using SPSS 25.0 (IBM, Armonk, NY, USA).

## Results

### Strain identification results

**FIGURE 1:**
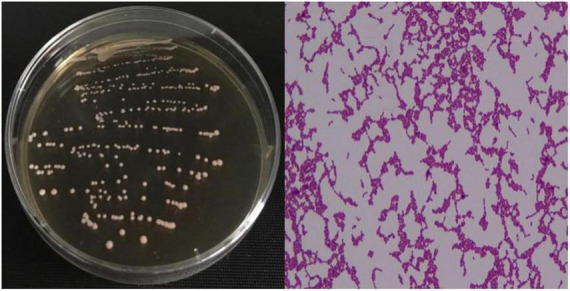
Colony morphology and Gram staining results of the strain.

The strain Lr-0601 isolated from yak yoghurt was observed by Gram staining, and 16s rDNA sequencing and biochemical identification were performed. As shown in Figure 1, strain Lr-0601 formed single colonies with consistent morphology on De Man, Rogosa, and Sharpe (MRS) medium agar plates, which were mostly white and round in morphology, with neat edges, and with moist and smooth surfaces. In addition, Gram staining of the strain revealed a short blue-purple rod with uniform cell morphology, which was determined to be a pure Gram-positive bacterium. Further 16s rDNA sequencing and biochemical identification showed that strain Lr-0601 was *Lacticaseibacillus rhamnosus* (Table 2).

**TABLE 2:** API 50 CH fermentation results of Lr-0601.

Carbohydrate name	Reaction result	Carbohydrate name	Reaction result
Negative control	−	Aesculus ferric citrate	+
Glycerol	−	Salicin	+
Erythritol	−	D-Cellobiose	+
D-Arabinose	−	D-Maltose	+
L-Arabinose	−	D-Lactose	+
D-Ribose	+	D-Melibiose	−
D-Xylose	−	D-Sucrose	+
L-Xylose	−	D-Trehalose	+
D-Ribitol	−	Inulin	−
Methyl-β-D Xylopyranoside	−	D-Matsutose	+
D-Galactose	+	D-Raffinose	−
D-Glucose	+	Modified starch	−
D-Fructose	+	Glycogen	−
D-Mannose	+	Xylitol	−
L-Sorbitose	+	D-Gentiobiose	+
L-Rhamnose	+	D-Toron sugar	+
Dulcitol	−	D-Lyxose	−
Inositol	−	D-Tagatose	+
Mannitol	+	D-Salalose	−
Sorbitol	+	L-Salalose	−
Methyl-α-D-Mannopyranoside	−	D-Arabinol	−
Methyl-α-D-Glucopyranoside	+	L-Arabinol	−
N-Acetyl Glucosamine	+	Potassium Gluconate	+
Amygdalin	+	Potassium 2-ketogluconate	−
Arbutin	+	Potassium 5-ketogluconate	−

### Acid and bile salt resistance

Gastric juice is considered one of the primary physiological challenges to be faced by probiotic strains due to the low pH and antimicrobial effects of pepsin, while the survival of probiotics in the small intestine is considered another challenge due to the presence of bile salts and pancreatic enzymes in the small intestine. Thus the ability of strains to tolerate bile salts of gastric juice can reflect whether the strains can survive the passage through the upper gastrointestinal tract after entering the host and thus reach the intestine to exert beneficial effects. Lr-0601 exhibited a considerably strong tolerance *in vitro* in simulated gastrointestinal environment (Table 3). Lr-0601 was still vigorously grown in the artificial gastric juice (pH 3.0), and growth rate reached 119%. The growth rate of Lr-0601 in bile salt solution decreased with increasing concentration but exhibited a higher growth ability in 0.3% bile salt solution.

**TABLE 3:** Effect of simulated gastrointestinal environment on viability of Lr-0601.

Strain	Survival rate in artificial gastric juice pH 3.0(%)	Growth rate in bile salts(%)		
		**0.10%**	**0.20%**	**0.30%**
Lr-0601	119.53 ± 1.12	77.97 ± 1.56	60.78 ± 1.39	41.64 ± 0.06

Results are shown as means ± SD. Lr-0601, *Lacticaseibacillus rhamnosus* 2016SWU.05.0601.

### Effect of *L. rhamnosus* 2016SWU.05.0601 on colon length and weight in mice with UC

**FIGURE 2:**
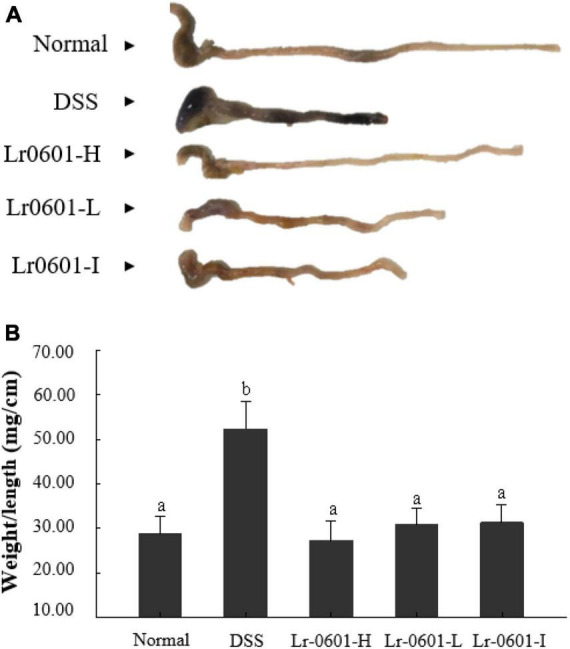
Morphological observation of colon in mice. **(A)** Colon length; **(B)** colon weight/length ratio. According to the Duncan’s multiple range tests, significant (*p* < 0.05) was found between groups labeled with different lowercase letters (a, b). There was no significant difference (*p* > 0.05) between the groups marked with the same lowercase letters (a, b). Normal, normal mice; DSS, mice treated with DSS; Lr-0601-H, high dose of*L. rhamnosus* 2016SWU.05.0601 (1.0 × 1010 CFU/kg body weight (bw)/day); Lr-0601-L, low dose of *L. rhamnosus* 2016SWU.05.0601 (1.0 × 109 CFU/kg bw/day); Lr-0601-I: heat-inactivated *L. rhamnosus* 2016SWU.05.0601.

DSS-induced colitis caused congestion and edema in the colon of mice, resulting in a significant increase in colon weight and a significant shortening of colon length (Figure 2A). The colon weight-length ratio is an important indicator of the severity of colitis, and in general, the higher the ratio is, the more pronounced the shortening and edema of the colon in the mouse, and the more severe the condition is. Compared with the normal group, the colon weight-length ratio of mice in the DSS group was significantly increased (*p* < 0.05), whereas after supplementation with different doses of Lr-0601 treatment, the shortening of the colon, edema and congestion were alleviated, and the colon weight-length ratio of mice was significantly decreased (*p* < 0.05). It is noteworthy that the colon weight-length ratio of mice in the Lr-0601-H group, was closer to that of the normal group (Figure 2B). Overall, it was demonstrated that Lr-0601 was effective in alleviating the adverse effects of DSS-induced colitis on the colon of mice.

### Effect of *L. rhamnosus* 2016SWU.05.0601 on colon histology in mice with ulcerative colitis

**FIGURE 3:**
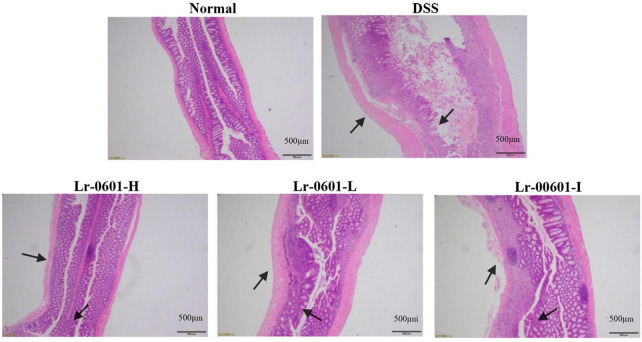
Histopathology observation of colon tissues in mice (magnification 100 × ). Representative inflammatory lesions and tissue damage in the colon are shown by arrows.

Tissue damage can be visualized by H&E staining. Upon observation of pathological histological sections of the mouse colon, we found that DSS mice exhibited significant inflammatory cell infiltration, mucosal damage and crypt loss, and significant oedema and ulceration of the colon were observed (Figure 3). In contrast, colonic damage was significantly reduced in mice receiving Lr-0601 intervention, and all three intervention conditions showed positive improvement.

### Effect of *L. rhamnosus* 2016SWU.05.0601 on serum cytokine levels in mice with ulcerative colitis

**FIGURE 4:**
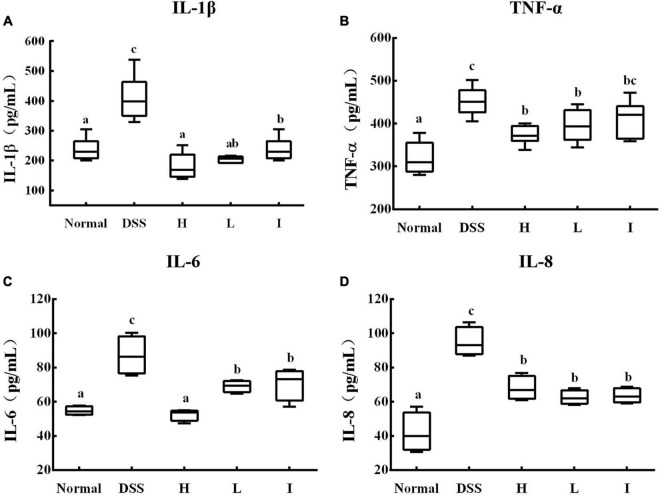
Levels of cytokines in serum of different experimental groups. **(A)** IL-1β cytokine level in serum; **(B)** IL-6 cytokine level in serum; **(C)** IL-8 cytokine level in serum; **(D)** TNF-α cytokine level in serum. a-c Mean values with different letters over the bars are significantly different (*p* < 0.05). Normal, normal mice; DSS, mice treated with DSS; H, high dose of *L. rhamnosus* 2016SWU.05.0601 (1.0 × 10^10^ CFU/kg body weight (bw)/day); L, low dose of *L. rhamnosus* 2016SWU.05.0601 (1.0 × 10^9^ CFU/kg bw/day); I, heat-inactivated *L. rhamnosus* 2016SWU.05.0601.

Serum inflammatory factor levels can reflect the level of body inflammation to a certain extent, so we detected several typical pro-inflammatory factors in serum, including IL-1β, IL-6, IL-8, and TNF-α. The results showed that compared with the normal group, the serum levels of IL-1β, IL-6, IL-8, and TNF-α in the DSS group of mice were significantly elevated (*p* < 0.05; Figures 4A-D), whereas supplementation with Lr-0601 was able to significantly reduce the levels of these inflammatory factors and effectively alleviate the inflammation of the organism.

### Effect of *L. rhamnosus* 2016SWU.05.0601 on mRNA expression of inflammatory regulatory genes and tight junction proteins in the colon of mice with ulcerative colitis

**FIGURE 5:**
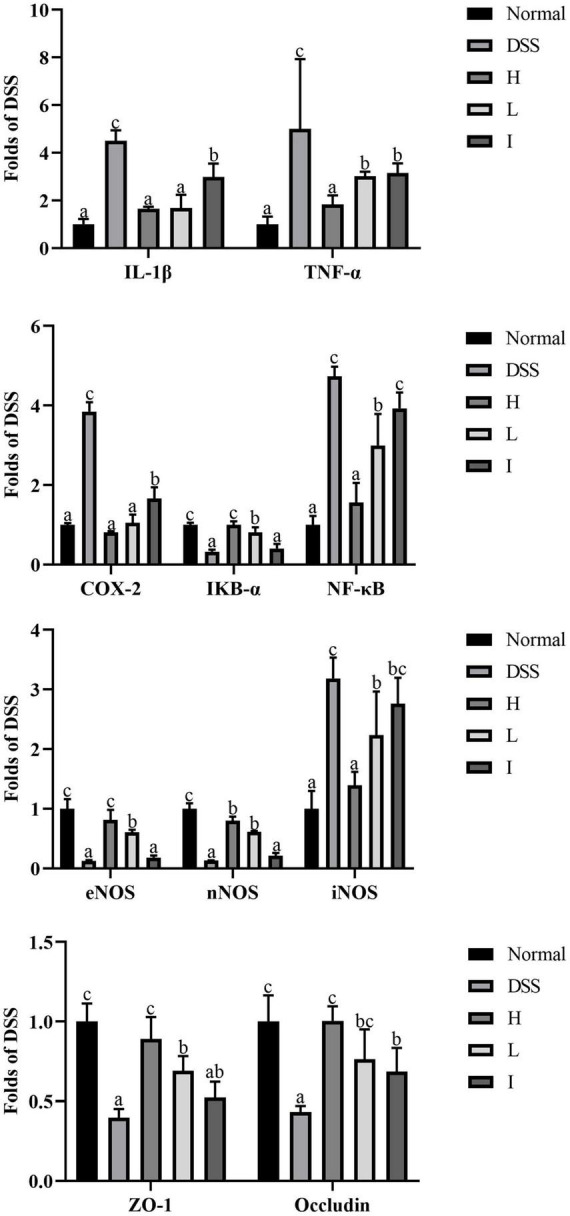
mRNA expression levels of TNF-α, IL-1β, eNOS, nNOS, iNOS, COX-2, IκB-α, NF-κB, ZO-1, and Occludin in colon tissues. a-c Mean values with different letters over the bars are significantly different (*p* < 0.05).

The intestinal barrier is the first line of defense to prevent harmful substances from entering the bloodstream, so the integrity of the intestinal barrier helps to maintain a normal intestinal micro-ecological balance and prevent the invasion of pathogens, which in turn serves to reduce inflammation and slow down disease progression. While the disruption of the intestinal barrier and body inflammation may be important triggers of ulcerative colitis, it was found that the expression of endothelial nitric oxide synthase (eNOS), neuronal nitric oxide synthase (nNOS), IκB-α, ZO-1 and Occludin was significantly reduced in the DSS group, while the expression of iNOS, COX-2, and NF-κB was significantly increased when compared with that of the normal group (*p* < 0.05) (Figure 5). In contrast, supplementation with Lr-0601 effectively reversed this phenomenon. Overall, the administration of Lr-0601 was able to repair the intestinal barrier damage and alleviate the disease by modulating the expression of key genes in the NF-κB-iNOS/COX-2 signalling pathway to reduce the body inflammation.

## Discussion

DSS model is widely used in animal models of UC because this method because of its simplicity and reproducibility. Pathological changes similar to UC in the human colon occurred when mice ingest DSS (Zhang et al., 2018). DSS-sensitized intestinal epithelial cells can directly contact immune cells, promote the production of reactive oxygen species, impair the intestinal barrier integrity, as well as increase the recruitment of immune cells and the production of inflammatory cytokines (Shin and Kim, 2018). In the animal experiments, DSS-treated mice showed intestinal flora imbalance, decreased microbial diversity and abundance, and changes in specific bacterial species (Taylor et al., 2022). As the role of gut flora in the etiology of ulcerative colitis is well established, microbiota modulation is an attractive area for applying methods such as probiotic supplementation to reduce inflammation and induce intestinal homeostasis. Lactic acid bacteria, comprising a common type of probiotics, have been used for the treatment of colitis with beneficial effects on intestinal health. However, the beneficial effects and mechanisms involved in different probiotics vary (Astó et al., 2019), so it is important to explore different probiotics for the alleviation of UC in order for them to facilitate their clinical use and increase the availability of treatments for UC patients.

In addition, probiotics are generally considered as living microorganisms that are beneficial to human health after appropriate doses are administered to humans (Astó et al., 2019). Therefore, the prerequisite for probiotics to function is to enter the human body in the form of live bacteria and a certain degree of colonization in the intestine. However, it has been found that the metabolites or cellular components of probiotics can also have a positive impact on the body, and this part is defined by ISAPP as postbiotics (Cai et al., 2022; Liu et al., 2022). Whereas previous studies have focused more on probiotics application to ameliorate UC and less on the role of postbiotics, in this study we investigated the effects of both probiotic and postbiotic applications for the treatment of UC. In this study, we evaluated the beneficial effects of live bacteria of strain Lr-0601 and its inactivated substances on DSS-induced UC mice.

We observed that Lr-0601 exerted a positive colon injury-relieving effect in mice after entering the body in a live bacterial state, with high-concentration Lr-0601 having a better effect. Interestingly, the inactivated form of Lr-0601 also showed beneficial effects on DSS-induced colitis, suggesting that postbiotics show similar potential to probiotics in alleviating colitis symptoms. Although the present study did not analyze the specific substances responsible for the beneficial effects of postbiotics, previous studies have shown that a variety of substances such as cell surface and other types of proteins, peptides, endo- and exopolysaccharides, extracellular vesicles, short-chain fatty acids, bacteriocins, enzymes, phospholipidic acids, peptidoglycan-derived polypeptides, vitamins, acetylated phospholipids, and organic acids, or their free supernatants (CFS) and many others, are beneficial for the body’s health, and that they have antimicrobial and immunomodulatory properties, and that they do not contain live microorganisms (Nataraj et al., 2020; Vallejo-Cordoba et al., 2020). These substances have antimicrobial, antioxidant and immunomodulatory effects, and their absence of live microorganisms, thus circumventing the risk of undesirable infections, are recommended for use in the treatment of UC.

Bacteriocins, cell wall and extracellular proteins from heat-inactivated probiotics were shown to reduce pro-inflammatory responses and inhibit cytokine-induced apoptosis in intestinal epithelial cells. In an animal study (Quévrain et al., 2016), a 15 kDa protein produced by Fusarium pratensis was identified to prevent UC by inhibiting the NF-κB signalling pathway in intestinal epithelial cells. Furthermore, in another cellular assay looking at the effects of heat-inactivated probiotics on UC, postbiotics were found to exert anti-inflammatory effects by inducing the secretion of IL-10 production by peripheral blood mononuclear cells from patients with UC, and inhibiting the secretion of IL-8 by HT-29 cells, thereby alleviating the effects of heat-inactivated probiotics (Imaoka et al., 2008). These substances such as bacteriocins, cell wall and extracellular proteins may also be the key components of Lr-0601 inactivator acting in this study and ameliorating UC by reducing inflammation.

Our results indicate that Lr-0601 and its postbiotic elements administered orally reduces the serum IL-1β, TNF-α, IL-6, and IL-8 levels in the DSS-induced UC model. These inflammatory cytokines play an important role in the development of UC. IL-1β and TNF-α can promote the infiltration of inflammatory substances by increasing the permeability of epithelial cells and endothelial cells, resulting in congestion, edema, and erosion of the colonic mucosa. In addition, aggregation of IL-1β and TNF-α stimulates the secretion of IL-6 and then activates the NF-κB signaling pathway (Neurath, 2022). IL-8 is the strongest chemokine, which aggravates inflammatory response by accumulating inflammatory cells to the colonic lesion (Chen et al., 2017). As a key transcription factor, NF-κB is widely distributed in various tissue cells and plays a key role in the occurrence and development of UC. In healthy state, NF-κB binds to its inhibitory protein IκB to form an inactive complex. In UC, IKK kinase induces phosphorylation of IκB and releases NF-κB from the complex. Free NF-κB then induces the production of inflammatory substances such as nitric oxide and prostaglandins (Li et al., 2020).

Simultaneously, NF-κB promotes the expression of downstream pro-inflammatory genes, such as IL-1β and TNF-α, which in turn further activate NF-κB, forming positive feedback regulation and expanding the inflammatory cascade (Shao et al., 2015; Dou et al., 2023). NO is an important inflammatory mediator in UC, and its synthesis is regulated by NOS. iNOS can continuously produce NO by catalyzing arginine, and eNOS and nNOS maintain NO synthesis balance (Hibiya et al., 2016). The inducible cyclooxygenase COX-2 is a key rate-limiting enzyme that induces arachidonic acid metabolism to produce prostaglandins (Hegazi et al., 2006). iNOS and COX-2 have binding sites for NF-κB; thus, their transcriptional expression is regulated by NF-κB. When NF-κB is overactivated in colon tissue, the activities of iNOS and COX-2 are correspondingly increased, inducing the production of large amounts of NO and PGE2. Excessive amounts of NO and PGE2 can dilate blood vessels and increase blood flow, causing congestion and edema of colon tissue. In the present study, Lr-0601 significantly inhibited the mRNA expression of NF-κB, iNOS, and COX-2 in the colon tissue of mice with UC as well as promoted the expression of IκB-α.

A complete intestinal barrier helps maintain intestinal homeostasis, and impaired intestinal barrier may contribute to intestinal disease. Tight junctions are the structural basis for the formation of a cell bypass seal, regulating intercellular molecular transport and maintaining selective permeability of intestinal epithelial cells. Therefore, tight junctions are key to protecting the intestinal mucosa from harmful substances in the external environment (Wan et al., 2019). ZO-1 is a peripheral membrane protein in tight junction proteins that links transmembrane proteins to the cytoskeleton. And Occludin is a transmembrane protein in tight junction proteins that regulate substance transport and seal cell by pass (Capaldo et al., 2017). In the UC animal model, DSS accumulated in the intestinal tract of mice impairs intestinal barrier integrity, improves intestinal permeability, and prompts the recruitment of immune cells and inflammatory cytokine production, disrupting intestinal homeostasis (Shin and Kim, 2018). We confirmed these results by measuring the mRNA expression of ZO-1 and Occludin. It was also observed that supplementation with Lr-0601 and its postbiotics significantly upregulated these tight junction proteins and protected the intestinal barrier function, which plays an important role in the prevention of UC.

**FIGURE 6:**
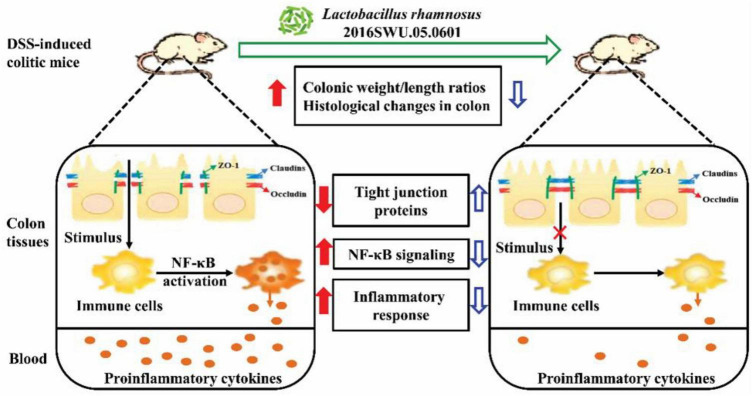
Protective effects of *L. rhamnosus* 2016SWU.05.0601 (Lr-0601) on dextran sulfate sodium (DSS)-induced colitis in mice. Lr-0601 increased the expression levels of tight junction proteins, which contributed to inhibit the activation of nuclear factor (NF)-κB signaling and inflammatory response in DSS-treated mice. These beneficial effects ultimately attenuated DSS-induced colitis. Red filled arrows represented changes in response to DSS; Blue unfilled arrows represented changes in response to DSS plus Lr-0601.

Overall, our study demonstrated that both Lr.0601 and its prepared postbiotics showed positive protection against UC, but the mechanism of their preventive effects on UC needs to be explored more deeply (Figure 6). In addition, we demonstrated the research and application potential of postbiotics in UC prevention, and compared with live probiotics, postbiotics are stable and biologically safe, they do not need to colonize the organism to exert beneficial effects after entering the organism, and it requires lower storage conditions than live bacteria, which makes it more suitable for diversified applications in different industries, but large-scale preclinical animal models and high-quality clinical studies in human are still needed to validate the safety and health effects of postbiotics. In conclusion, our findings highlight the beneficial effects of probiotics and postbiotics in UC remission and provide insights into the application of probiotic and postbiotic products in the prevention of UC, especially the multiple applications of postbiotics may be an effective complement to probiotics, which has a great potential as an extension of the direction of probiotics to provide a promising therapeutic strategy for live adjuvant therapies in the treatment of UC.

## Data availability statement

The original contributions presented in the study are included in the article/supplementary material, further inquiries can be directed to the corresponding author.

## Ethics statement

The animal study was approved by the Animal Care and Use Committee of Southwest University. The study was conducted in accordance with the local legislation and institutional requirements.

## Author contributions

LB: Data curation, Investigation, Writing – original draft, Writing – review & editing. YL: Data curation, Investigation, Writing – original draft, Writing – review & editing. CW: Resources, Writing – review & editing. YJ: Resources, Writing – review & editing. HS: Conceptualization, Funding acquisition, Project administration, Supervision, Visualization, Writing – original draft, Writing – review & editing.
